# TRP channels associated with macrophages as targets for the treatment of obese asthma

**DOI:** 10.1186/s12944-024-02016-0

**Published:** 2024-02-17

**Authors:** Wenzhao Zhu, Dinxi Bai, Wenting Ji, Jing Gao

**Affiliations:** Chengdu University of Traditional Chinese Medicine, 1166 Liutai Avenue, Wenjiang District, Chengdu, Sichuan China

**Keywords:** Obesity, Asthma, Obese asthma, TRP channels, Inflammation

## Abstract

Globally, obesity and asthma pose significant health challenges, with obesity being a key factor influencing asthma. Despite this, effective treatments for obese asthma, a distinct phenotype, remain elusive. Since the discovery of transient receptor potential (TRP) channels in 1969, their value as therapeutic targets for various diseases has been acknowledged. TRP channels, present in adipose tissue cells, influence fat cell heat production and the secretion of adipokines and cytokines, which are closely associated with asthma and obesity. This paper aims to investigate the mechanisms by which obesity exacerbates asthma-related inflammation and suggests that targeting TRP channels in adipose tissue could potentially suppress obese asthma and offer novel insights into its treatment.

## Introduction

Asthma, a chronic respiratory ailment, has become a pressing global health issue. In 2019, the prevalence of asthma among individuals aged 20 and over in China reached 4.2%, amounting to 45.7 million affected people. Generally, asthma is classified into various phenotypes, such as allergic asthma, late-onset asthma, and obesity-related asthma, allowing for personalized treatment approaches to achieve optimal therapeutic outcomes [1]. Among these, obesity-related asthma has garnered significant attention. Pertinent research indicates that obesity is a crucial factor in asthma, as alterations in adipose tissue function in patients with obesity-related asthma can trigger a systemic inflammatory state and a surge in both anti-inflammatory and pro-inflammatory cytokines [2]. Pertinent cytokines and adipokines, such as leptin and adiponectin, have the potential to influence alterations in cytokines, including Th1 and Th2 cytokines, as well as inflammatory cells. This is believed to be a crucial mechanism by which obesity exacerbates asthma. The prevalence of obese asthmatic patients has risen markedly in recent years, with a significant proportion of these individuals experiencing more severe symptoms than those with typical asthma [3]. Unfortunately, the systemic inhalation of high-concentration cortisol and other medications has proven to be insufficient for effectively controlling obese asthma, indicating that current asthma treatment approaches may not be suitable for this patient population [4]. As such, exploring the underlying connection between obesity and asthma and implementing targeted weight reduction strategies could potentially serve as an effective means of treating obese asthma.

The transient receptor potential (TRP) channel, a type of channel protein, is extensively distributed throughout the central nervous system and the non-nervous system. It plays a crucial role in regulating temperature, pressure, and vision, as well as modulating the Ca^2+^ plasma signal. TRP channels are categorized into seven distinct subfamilies: TRPC (classical), TRPV (vanilloid), TRPM (melastatin), TRPN (NOMPC-like), TRPA (ankyrin), TRPP (polycystin), and TRPML (mucolipin) [5]. Due to their widespread presence in various cells and their marked responsiveness to diverse stimuli, TRP channels have emerged as a focal point in targeted therapy research [6].

TRP channels are present in adipose tissue and are particularly abundant in immune cells. These channels are responsible for controlling fundamental heat production processes, as well as the secretion of cytokines and inflammatory factors. Consequently, our research is centred on TRP channels within adipose tissue. Through in-depth analysis, we discovered that cold exposure and ion signalling can modulate adipose tissue heat production via TRP channels [7]. The advantage of treating obese asthma through the TRP channel lies in starting from the inflammatory mechanism of obesity-induced asthma, rather than simply treating asthma. Compared to others, the TRP channel approach fundamentally improves obese asthma and provides new ideas for future drug development. Furthermore, by regulating TRP channels in immune cells, we may potentially curb obesity and alleviate symptoms in obese patients with asthma at their source. This approach could bridge the gap in current obese asthma treatment strategies and offer an innovative target for the evolution of therapeutic drugs.

## Introduction to obese asthma

Obesity and asthma, two pervasive diseases, exist both independently and interconnectedly. There is a discernible pattern that asthma is more prevalent within the obese population, with a significant percentage of people living with asthma also classified as obese. Numerous meta-analyses and cross-sectional studies corroborate the fact that obesity correlates with an over 50% increase in asthma prevalence in children. In addition, the prevalence rates among lean adults and obese adults stand at 7.1% and 11.1%, respectively, signifying the severity among obese individuals. Moreover, asthma of this nature is more challenging to control [1, 8], engenders higher treatment costs, and generally results in subpar treatment outcomes [9, 10].

A comprehensive cross-trait genome-wide association study involving 457,822 individuals established a substantial positive genetic correlation between obesity and late-onset asthma (defined by the age of onset of asthma) in subjects aged 16 years and above. A cross-trait meta-analysis identified 34 common loci amid three obesity-related traits and two asthma subtypes, providing robust support for the relationship between obesity and asthma [11].

In 2000, the severe asthma study (SARP) first employed the term “phenotype” for asthma categorization, a critical aspect of personalised clinical treatment [12]. Since then, “obese asthma” has been recognized as a distinct asthma phenotype, sparking several interrelated studies examining the mechanisms behind obesity-induced asthma and effective treatment strategies.

One significant investigation explored the aftermath of weight loss surgery in asthmatic patients. They discovered that post-surgery asthma improvements were associated with a marked reduction in the relative abundance of fecal matter, laxospirochetes, rosella, and verona (FLVR) in the gut flora and the prevalence of *Streptococcus pneumoniae*, *Haemophilus influenzae*, and *Moraxella catarrhalis* in the respiratory tract. The governing mechanism primarily entailed the induction of neutrophil-related inflammation and the eradication of allergenic effects to alleviate asthma symptoms. Moreover, weight loss surgery has demonstrated improvements in adipose tissue dysfunction in obese patients [13, 14].

In a prospective study of 14 asthmatic patients following bariatric surgery, there was a reduction in the usage of inhaled steroids, with the median equivalent dose of beclomethasone decreasing from 460 μg at baseline to 218 μg one year after surgery [15].

As for the genesis of obesity-induced asthma, apart from considering factors such as obesity’s impact on lung function and mechanics, the alteration of airway diameter, decrements in airflow, and shifts towards a procoagulant airway state [16], we should also regard obesity as a systemic low-grade inflammatory disease. The systemic inflammatory response triggered by obesity may be a significant contributor to the development of asthma [17, 18].

### Mechanism of obese asthma

The tight connection between obesity, obese asthma, and adipose tissue is well established [19]. Adipose tissues in mammals can be broadly divided into two categories: white adipose tissue (WAT) and brown adipose tissue (BAT) [20]. The primary role of WAT is to store surplus energy within the body as triglycerides. Previously, it was inferred from early studies that BAT was predominantly present in rodents and newborns [21], with a rare occurrence in adults and seemingly no direct correlation with caloric combustion. However, a sequential 18 F-fluorodeoxyglucose (18 F-FDG) positron emission tomography and computed tomography (PET-CT) study revealed a considerable amount of BAT in adults, specifically from the neck to the chest. This finding lends credence to the important role BAT plays in energy expenditure and fat conversion. Consequently, this suggests that BAT has a significant relationship with obesity and holds the potential for obesity treatment [22]. BAT’s functionality comprises the dissolution of oxidative phosphorus in the mitochondrial oxidative respiratory chain via a mitochondrial protein called uncoupling protein 1 (UCP1). This process inhibits the synthesis of adenosine triphosphate (ATP) while converting electrochemical energy into heat [23, 24], thereby serving to release energy and generate heat.

In obese patients, adipose tissue is involved in energy consumption and fat conversion. As weight increases, the adipose tissue itself responds to inflammatory cells and stimuli, releasing more adipokines through activated white blood cells and other mediators [25]. Due to the presence of neutrophil inflammation and the increased inflammatory response caused by AT, obese asthma patients often develop more severe asthma [26]. According to previous studies, obese subjects with asthma have more leptin and less adiponectin than those with asthma. In asthma patients, leptin induces inflammation of lung fibroblasts by enhancing the production of further pro-inflammatory chemokines and cytokines. When patients develop leptin resistance, these factors seem to be inhibited to some extent [27, 28]. In contrast, adiponectin can have anti-inflammatory effects in airway cells by promoting the release of anti-inflammatory cytokine IL-10 and inhibiting airway inflammation. Immune cells around adipose tissue, especially macrophages, secrete Tumor necrosis factor (TNF) and IL-1β. Changes in the number of cytokines that reach the lungs through blood circulation can play a pro-inflammatory role, causing airway hyperresponsiveness and ultimately leading to airway inflammation, leading to asthma. Therefore, how to control the production of cytokines and whether there are targets for adipose tissue and immune cells to regulate their production of cytokines may become a noteworthy part of controlling the onset of obese asthma [27].

### Obese asthma classification

In addressing obese asthma, unveiling the mechanisms through which obesity culminates in asthma is critical. Obese asthma can largely be divided into two phenotypes: one is dyspnea resulting from the collapse of pulmonary airways and trachea due to obesity, and the other mirrors allergic asthma associated with immune system disorders induced by obesity. Just as with asthma, obesity—which is a chronic inflammatory disease—can instigate a state known as “meta-inflammation” throughout the body [29]. Particularly significant is the role adipose tissue, specifically BAT, plays in relation to asthma inflammation [30]. BAT not only produces a multitude of cytokines but also influences the production of adipokines. This suggests that inflammation may potentially serve as a key nexus between obesity and asthma.

#### Related immune cells

Adipose tissue houses an abundance of immune cells that both influence and interact with its inflammatory state. Among these, macrophages play a pivotal role [31]. In the ensuing discussion. We will delve into the mechanisms by which macrophages partake in the inflammatory processes within adipose tissue, as well as their links to obesity and asthma. Furthermore, we will shed light on the functions of other immune cells and delineate their interactions with macrophages.

##### Macrophages

Macrophages are bifurcated into M1 and M2 subtypes [32], existing across a spectrum and with most macrophages occupying an intermediate state [33]. In individuals with normal body weight, the resident macrophages in BAT are predominantly of the M2 subtype. An accumulation of M2 macrophages aids in reducing inflammation in adipose tissue. However, in obese individuals, BAT is infiltrated by an influx of M1 macrophages due to a sustained inflammatory state [34, 35]. M2 macrophages secrete the cytokine IL-10, which helps attenuate inflammation and maintain the metabolic homeostasis of adipose tissue. In contrast, M1 macrophages in the adipose tissue of obese individuals release pro-inflammatory cytokines, such as IL-17 and IFN-γ, thereby escalating inflammatory responses [36]. Relevant research, conducted by processing the adipose tissue of obese adult patients after weight-loss surgery and quantifying M1 and M2 macrophages using flow cytometry, found that the M1 macrophage content was higher in the adipose tissue of obese asthma patients. Moreover, it was observed that disease severity in obese asthma patients directly correlated with the percentage of M1 macrophages present in adipose tissue, highlighting the inflammatory relationship between macrophages and obese asthma [37]. Given the experiment’s limited sample size and the observation that obesity has a more pronounced impact on asthma in women than in men, we postulate that increasing the sample size and controlling for gender disparities may yield even more compelling results.

Numerous infiltrating macrophages commonly converge around inflamed BAT, leading BAT to curtail thermogenesis and activate mitochondrial uncoupling protein 1 (UCP1) [38]. Classically activated M1 macrophages release high levels of pro-inflammatory cytokines, including TNF-α, MCP-1, IL-6, and IL-1β. Conversely, alternatively activated M2 macrophages generate anti-inflammatory cytokines, such as interleukin 4 (IL-4) [39]. Adipose tissue abnormalities in obese patients trigger the dysregulated secretion of several substances, including IL-6, TNF-α, MCP-1 [40], and an array of adipokines, such as leptin and adiponectin (ADPN) [17]. The airway inflammation present in most asthmatic patients is linked with an increase in Th-2 type cytokines, leading to eosinophil accumulation within the airway wall and the subsequent overproduction of mucus. This process results in severe airway inflammation [41, 42]. Many of these factors are complicit in driving airway inflammation associated with obesity and asthma.

##### Other immune cells

In addition to macrophages, other immune cells participate in the inflammatory process in obese asthma. On the one hand, they act alone; on the other hand, they interact with macrophages [43]. There is also a state called obesity-associated inflammation in obese tissue, which causes immune cells in obese tissue to aggregate and release multiple pro-inflammatory factors.

One of these immune cells is mast cells (MCs), which are also innate immune cells that are widely distributed in adipose tissue. They originate from CD34^+^, CD13^+^, and CD17^+^ pluripotent hematopoietic stem cells and are related to macrophages [44]. MCs are mainly distributed between adipocytes and near blood vessels. Reducing MCs can help inhibit the infiltration of M1 macrophages and reduce the pro-inflammatory cytokines TNF-α and MCP-1 and other substances that can alleviate the inflammatory state of adipose tissue cells [45]. In addition, MCs are directly related to adipose tissue heat production by promoting the generation of adipose tissue cells. The increase in MCs is accompanied by the increase in serum proteases, which inhibit the expression of adiponectin and have a negative effect on the heat production of adipose tissue [46]. These findings indicate that MCs not only interact with macrophages but also participate in the inflammatory mechanism of obesity alone.

The presence and function of ILC2 in adipose tissue are regulated by IL-33. ILC2 secretes IL-5 and IL-13 to maintain the normal level of eosinophils in adipose tissue, which is essential for normal heat production [47]. In obese mice, a reduced level of IL-33 leads to a lower expression of ILC2. IL-5 is a key cytokine for ILC2 and is responsible for sustaining the eosinophil and M2 populations that contribute to adipose tissue thermogenesis [48]. It has been suggested that stimulating eosinophils and M2 through the ILC2-IL-5 axis may be effective in counteracting adipose tissue inflammation, as eosinophils can prevent obesity and insulin resistance [49]. This demonstrates the importance of ILC2 in adipose tissue.

T cells can produce inflammatory factors, such as interferon-γ (IFN-γ), to modulate the inflammation of adipose tissue. The most crucial role of various T cells is to regulate obesity inflammation by affecting macrophages. Inflammatory T cells, such as Th1 and Th17, can release IFN-γ and IL-17 to activate pro-inflammatory macrophages [50]. On the other hand, anti-inflammatory T cells, such as Th2 and Foxp3 + Treg, can induce macrophages to differentiate into anti-inflammatory macrophages by secreting IL-4 and IL-13 [51].

A study using mice with a transgenic that encodes Cre-rominase under the control of the NK cell-specific NKP46 promoter and a transgena that allows diphtheria toxin (DT)-mediated depletion of Cre-expressing cells found that NK cell parting was associated with reduced macrophage infiltration in visceral adipose tissue and that NK cell emptying could improve the insulin sensitivity of obese mice [52]. In addition, adipocyte-derived ligands for NK cell-activating receptors (NCR1) can stimulate NK cell proliferation and IFN-γ production, which improves ATM activation and insulin resistance in obesity [53].

It can be found in various immune cells that the inflammatory process of obese asthma is closely relevant to adipokines and cytokines. Among various factors, leptin, adiponectin, and several cytokines contribute to the inflammatory process of obesity and asthma and are regulated by or affect the function of immune cells. They may be involved in the mechanism of obesity and asthma, which is of discussion value.

#### ADPN and leptin

The inflammatory state induced by obesity affects the secretion of adipokines by adipose tissue. Leptin and adiponectin are also involved in airway hyperresponsiveness and inflammation. Leptin can aggravate airway hyperresponsiveness, while adiponectin can alleviate it [43]. Therefore, they deserve more attention.

ADPN is a 244-amino acid protein secreted by adipose tissue and widely expressed in various cell types, including human and mouse osteoblasts, liver cells, muscle cells, epithelial cells, and placental tissue. In adipocytes, ADPN increases glucose uptake and stimulates fatty acid oxidation [54]. Obese patients have reduced ADPN secretion from adipose tissue. Adiponectin can induce lung macrophages to produce IL-4 and increase the expression of TNF-α, IL-6, and IL-12, which play pro-inflammatory roles. In human monocyte-derived macrophages, transcriptome analysis shows that adiponectin promotes the differentiation of M1. The effect of adiponectin on monocyte-macrophage cytokine production has been demonstrated in unstimulated or (Lipopolysaccharide) LPS-stimulated preparations, which play pro-inflammatory roles [55].

Leptin is a 167-amino acid protein mainly produced by adipocytes and macrophages in adipose tissue. It can promote triglyceride breakdown, inhibit fatty acid synthase expression, and exert significant pro-inflammatory effects. Leptin-deficient mice have reduced allergic airway inflammation symptoms, and asthmatic patients have serum leptin levels that are generally 50% higher than those of non-asthmatic patients [56]. Leptin-treated macrophages may accumulate lipid droplets, which may facilitate macrophage inflammation. In addition, leptin can enhance macrophage activation induced by LPS and IL-4, and activated macrophages in adipose tissue can release TNF-α and IL-6, which can interfere with insulin signalling in adipocytes, disrupt glucose metabolism, impair immune metabolism, and cause systemic low-grade inflammation in obese patients [57]. Leptin has structural similarities with cytokines, such as IL-6 and IL-11, which suggests that leptin may have immunomodulatory effects [42]. Leptin can stimulate the secretion of pro-inflammatory cytokines, such as TNF-α and IL-6 [58].

Besides the traditional adipokines, some newly discovered adipokines such as omentin [59], a novel hydrophilic adipokine, have an inverse correlation with obesity and a positive correlation with ADPN levels. One study found that omentin mRNA increased 9.5-fold in airway epithelial cells in asthmatic patients [60], and they hypothesized that omentin may be involved in asthma-induced airway inflammation, airway hyperresponsiveness, and mucus overproduction.

Adipose factors are produced by AT and take part in airway inflammation. The secretion of these adipose factors is related to obesity. If obesity can be suppressed and inflammation in adipose tissue can be reduced by activating some channels in adipose tissue and surrounding cells, it is possible to control the secretion of adipose factors, thereby inhibiting obese asthma.

#### Th2-type cytokines

Th2-type cytokines are involved in the pathogenesis of airway inflammatory diseases such as asthma. The most prominent are IL-4, IL-5, IL-6, IL-10, and IL-33 [17, 41, 61]. IL-4, produced by ILC2 cells and eosinophils [35], induces the expression of two anti-inflammatory cytokines by M2, TGF-β, and IL-10 [62]. IL-4 helps maintain the stability of M2 macrophage numbers in adipose tissue. Transforming growth factor-β (TGF-β) and IL-10 preserve the insulin sensitivity of adipocytes and regulate the energy expenditure by adipocytes. TGF-β also inhibits adipogenesis [63]. The level of IL-5 in the sputum of obese asthmatics was significantly higher than that of leaner asthmatics, suggesting that the amount of IL-5 cytokines is associated with obese asthma.

IL-6 has been shown to be linked to obese asthma. One study observed a significant relationship between IL-6 at baseline and the risk of asthma exacerbations requiring systemic corticosteroid therapy. For each quartile increase in serum IL-6, the risk of experiencing at least one acute exacerbation increased by 24%. Individuals with increased IL-6 levels have reduced lung function and an increased risk of exacerbations, and IL-6 increases with an increasing BMI [64]. It may also be related to a new asthma phenotype, which is identified and characterized by IL-6TS-specific epithelial gene markers rather than type 2 (T2) inflammation in the lung epithelium [65]. Similar to IL-5, obese asthmatics have higher levels of IL-6 in the peripheral blood than regular asthmatics. In humans, IL-33 can counteract excessive inflammation in AT by targeting immune cells that express ST2 receptors, but low serum IL-33 levels often accompany a high BMI. The two isoforms of ST2 identified so far are the full-length receptor (ST21) and the soluble ST2 (Sst2). Sst2 acts as a decoy receptor and can block IL-33 signalling [66]. Related studies have found increased obesity and worsened metabolic profiles in mice lacking ST2 or IL-33. IL-33 triggers the expansion of a subset of Fox3 + ST2 + T cells while attenuating adipose tissue inflammation [67]. IL-33 can activate eosinophils and maintain the stability of M2 macrophages, which can secrete IL-10, a cytokine that helps dampen inflammation and maintain metabolic homeostasis in adipose tissue. ILC2 cells can produce IL-5, a cytokine necessary for eosinophil expansion and survival. IL-33 is a key inducer of ILC2, and ILC2 in WAT can promote WAT browning and help prevent obesity development. However, its specific mechanism still requires further research.

It is clear that there is a connection between adipokines and cytokines, and the effect of adipokines varies in different macrophages. ADPN induces the expression of pro-inflammatory cytokines (IL-6, TNF-α, and IL-12) in M1, while in M2, it induces IL-10 and IL-1 receptor antagonists [68]. Leptin can support the proliferation and survival of ILC2 and Th2 cells, stimulating the production of IL-4, IL-5, and IL-13. Its interaction with IL-33 can also enhance airway inflammation and induce obesity and asthma [69].

Obese asthmatics are of interest mainly because they do not respond well to conventional treatments for asthma [70, 71] or because these treatments have some therapeutic risks. Some studies have detected increased biomarkers of glucocorticoid (GC) insensitivity in the lungs and peripheral blood of overweight asthma patients, making inhaled glucocorticoid therapy ineffective for treating obese asthma patients. Some therapies aimed at weight loss, such as exercise, diet control, and other interventions [14, 72], can achieve weight loss [73], but they are slow and long lasting. Therefore, more direct therapies are required [74]. Bariatric surgery is more promising, as it can lower airway inflammatory markers and enhance lung function [75] as a potential treatment for obese asthma, but it is not suitable for widespread use and clinical trials due to the small sample size and lack of control groups in relevant trials. Some pharmacological treatments are also attracting attention, such as the use of antidiabetic drugs: metformin and glucagon-like peptide 1 agonists can reduce obesity and inflammation [76] and are also regarded as a possible way to treat obese asthma, but their effect is not significant. Vitamin D has been shown to be related to both obesity and asthma, and vitamin D supplementation has a therapeutic effect in children with obese asthma [77].

Some of the potentially effective treatments for obese asthma have limitations, and side effects and complications may limit their use, in addition to the fact that most patients with obese asthma are severely ill. The high cost is also a real factor to consider. Thus, we need to find a more targeted treatment for obese asthma [78]. Through our discussion of the inflammatory mechanisms of obesity and asthma, we have found valuable macrophages and other related immune cells involved in this process. Starting with immune cells, we hope to find suitable targets in immune cells to control heat production. Among various potential targets, TRP channels are chosen to further elaborate on intervention in the inflammation of obese asthma by highlighting interactions between them to illustrate the feasibility of the treatment of obese asthma.

## TRP and obese asthma

In 1969, a groundbreaking study used methyl methanesulfonate (EMS) to induce mutations in fruit flies (*Drosophila melanogaster*) and screened for new mutants. One of the mutants discovered exhibited abnormal phototaxis and retinal function, as sustained light stimulation failed to elicit changes in retinal membrane potential. This mutant was named TRP [79]. TRP channels are widely distributed in both the nervous and non-nervous systems of various organisms and play a crucial role in sensory responses. Currently, TRP channels have seven subfamilies: TRPC, TRPV, TRPM, TRPN, TRPA, TRPP, and TRPML [80]. Some of these subfamilies are expressed in adipose tissue. TRP channels are primarily involved in mediating cold exposure and the activation of dietary compounds, such as capsaicin, tea catechins, and ephedrine [81]. They also contribute to energy metabolism and regulation in adipose tissue [82]. Therefore, the TRP channel can achieve the purpose of reducing the physical pressure of the airway and alleviating the inflammatory state of obese asthma to assist in the treatment of obese asthma. TRP is also closely related to adipokines and cytokines that correlate with obese asthma. Some TRP channels are involved in adipokines and cytokines secretion process and mediate the inflammation of obese asthma. In view of the correlation between TRP channels and obese asthma, TRP is likely to become an effective target for the treatment of obese asthma.

**Fig. 1:**
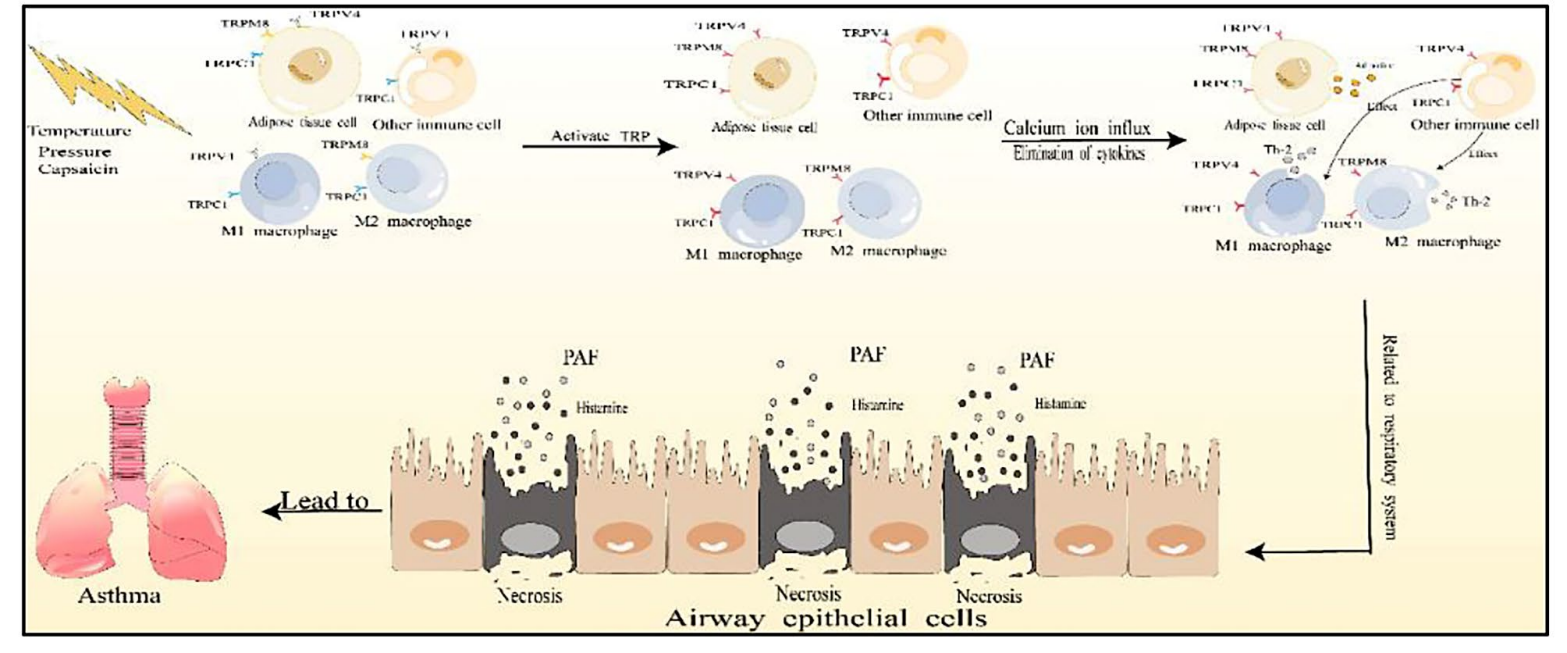
The inflammatory mechanisms of asthma related to TRP channels. This figure mainly describes the influence of fat cells and their surrounding immune cells on the inflammatory state of asthma when the TRP channel is activated, and this inflammatory state builds a bridge between obesity and asthma

## Various TRP channels and obese asthma

According to existing studies, a significant number of TRP channels have been found to be associated with obese asthma. Some of these channels play a role in generating heat in adipose tissue, while others are involved in airway inflammation, such as asthma. For instance, TRPV2 is highly shown off in BAT, and when TRPV2 is knocked out, the display of heat genes in BAT decreases [83]. Additionally, certain air pollutants, such as dust and nitrogen compounds, can activate TRPA1 and TRPV1, promoting and worsening existing asthma [84]. Due to the high expression of certain TRP channels, particularly in immune cells like macrophages, and the findings from previous research, as well as their clear or potential involvement in the inflammatory process of obese asthma, we have specifically chosen to discuss TRPV4, TRPM8, and TRPC1. These channels are primarily associated with obese asthma through their role in inflammation, and they are also linked to the production of fat and inflammatory factors (Fig. 1). In the future, they may play a vital role in the therapy of obese asthma.

### TRPV4

TRPV4, a member of the TRPV family, is a channel that can be activated by pressure, temperature, shear, and mechanical stimuli. It plays a crucial role in adipogenesis and various mechanisms associated with adipose tissue function, as it is highly expressed in adipose tissue. TRPV4 is expressed in various immune cells and actively participates in inflammatory processes, which aids in regulating adipose tissue heat production. This indicates its involvement in maintaining the physiological balance of AT [85]. TRPV4 is also involved in asthma. Studies have shown that TRPV4 can mediate the differentiation of lung fibroblasts and contribute to airway remodelling by activating a novel oxidase [86]. We will discuss the role of TRPV4 in the inflammatory process, especially in macrophages, to demonstrate the importance of TRPV4 in the inflammatory process of obese asthma and the feasibility of targeting TRPV4 in the treatment of obese asthma.

TRPV4 is widely distributed in the immune cells surrounding adipose tissue, with a prominent presence in macrophages. Its main role is to regulate the number and status of immune cells, thereby influencing the secretion of various cytokines. Specifically, TRPV4 mediates phagocytosis stimulated by bacterial lipopolysaccharide (LPS) and downregulates pro-inflammatory cytokines in macrophages [87]. In synovial cells, hypotonic stimulation activates TRPV4 channels, leading to the production of reactive oxygen species (ROS). As a key intracellular mediator for activating pro-inflammatory signalling pathways, ROS promotes the polarization of M1 macrophages. In a rat model of osteoarthritis induced by radial medial meniscus transection, intraarticular administration of HC067047, a selective TRPV4 inhibitor, decreased the M1 polarization of synovial macrophages and resulted in reduced synovial inflammation, cartilage degradation, and osteophyte formation. In addition, blocking TRPV4 through the ROS/NLRP3 signalling pathway reduces M1 macrophage polarization [88]. TRPV4 also negatively regulates PPARγ in adipocytes, affecting the expression of mitochondrial uncoupling protein 1 (UCP1) and cellular respiration. PPARγ is a vital transcription element in M2 macrophages and is involved in fatty acid uptake and oxidation [89]. Furthermore, a study utilizing immunofluorescence and digital calcium imaging technology analysed the interaction between the calcium-sensitive receptor CaSR and the TRPV4 channel, revealing their ability to promote Ca^2+^-dependent M1 macrophage polarization through the PLA2/CYP450 and PLC/PKC pathways [90]. Overall, TRPV4 is crucial in macrophage polarization and the secretion of cytokines associated with inflammation.

Asthma is primarily characterized as an inflammatory disease affecting the airways. TRPV4 is expressed in goblet cells and ciliated cells within the respiratory tract and plays a crucial role in airway inflammation, including asthma. In fibroblasts, TRPV4 functions as a calcium channel, mediating the formation and activation of fibroblasts. This channel also assists the endothelium in protecting the lungs from inflammation and infection, while enhancing lung barrier function [91]. Research has demonstrated the ability of TRPV4 to mediate the differentiation of lung fibroblasts and induce airway remodelling through the activation of novel oxidases [86]. Furthermore, studies involving mice grouping have revealed that TRPV4 integrates TGFβ and ROS signalling via NOX4. The interaction between TRPV4 and NOX4 can be targeted to mitigate lung remodelling associated with asthma [42]. However, a study investigating the repeated sensitization of wild-type (WT) and TPRV4 knockout (KO) mice with chicken ovalbumin (OVA) and repeated aerosol exposure to 1% OVA found no meaningful difference in the development of allergic asthma between the two arrays. It is significant to note that this study solely relies on eosinophil levels as a measure of asthma severity, which may introduce bias. Nevertheless, the overall consensus supports the universal role and significance of TRPV4 in inflammatory diseases, including asthma.

In addition to macrophages, TRPV4 is also expressed in adipocytes and other immune cells. The activation of TRPV4 has an impact on the secretion of adipokines in adipose tissue. Specifically, the activation of TRPV4 leads to an increase in leptin production, while the knockdown of TRPV4 inhibits leptin secretion. Interestingly, the effect of TRPV4 on lipocalin is opposite to that of leptin. Changes in leptin and ADPN levels have been found to be associated with allergic airway responses and to serve as key substances linking obesity and asthma [92]. These findings highlight the potential of TRPV4 channels in the treatment of obese asthma. Perhaps activating the TRPV4 channel on adipocytes or immune cells can reduce the secretion of leptin and adiponectin, prevent the content of adipocytokines in the surrounding blood, inhibit the occurrence of airway hyperresponsiveness, and ultimately achieve the goal of controlling asthma. Furthermore, one study discovered that TRPV4-deficient adipocytes exhibit higher ADPN production, while TRPV4-deficient 3T3-L1 adipocytes show reduced levels of leptin. In fact, the protein composition of adiponectin and leptin is altered by more than 50% in TRPV4-deficient cells [93]. The flow of Ca^2+^ is relevant to T cell proliferation, differentiation, and cytokine secretion, and TRP channels play a decisive role as ion channels in this process. In T cells, TRPV4 and TRPV1 can form heterodimeric channels that work synergistically to control the influx and efflux of Ca^2+^. The increase in heterodimeric channels is associated with T cell proliferation and the production of IFN-γ and IL-2, suggesting that TRPV4 takes part in the immune response of T cells.

**Fig. 2:**
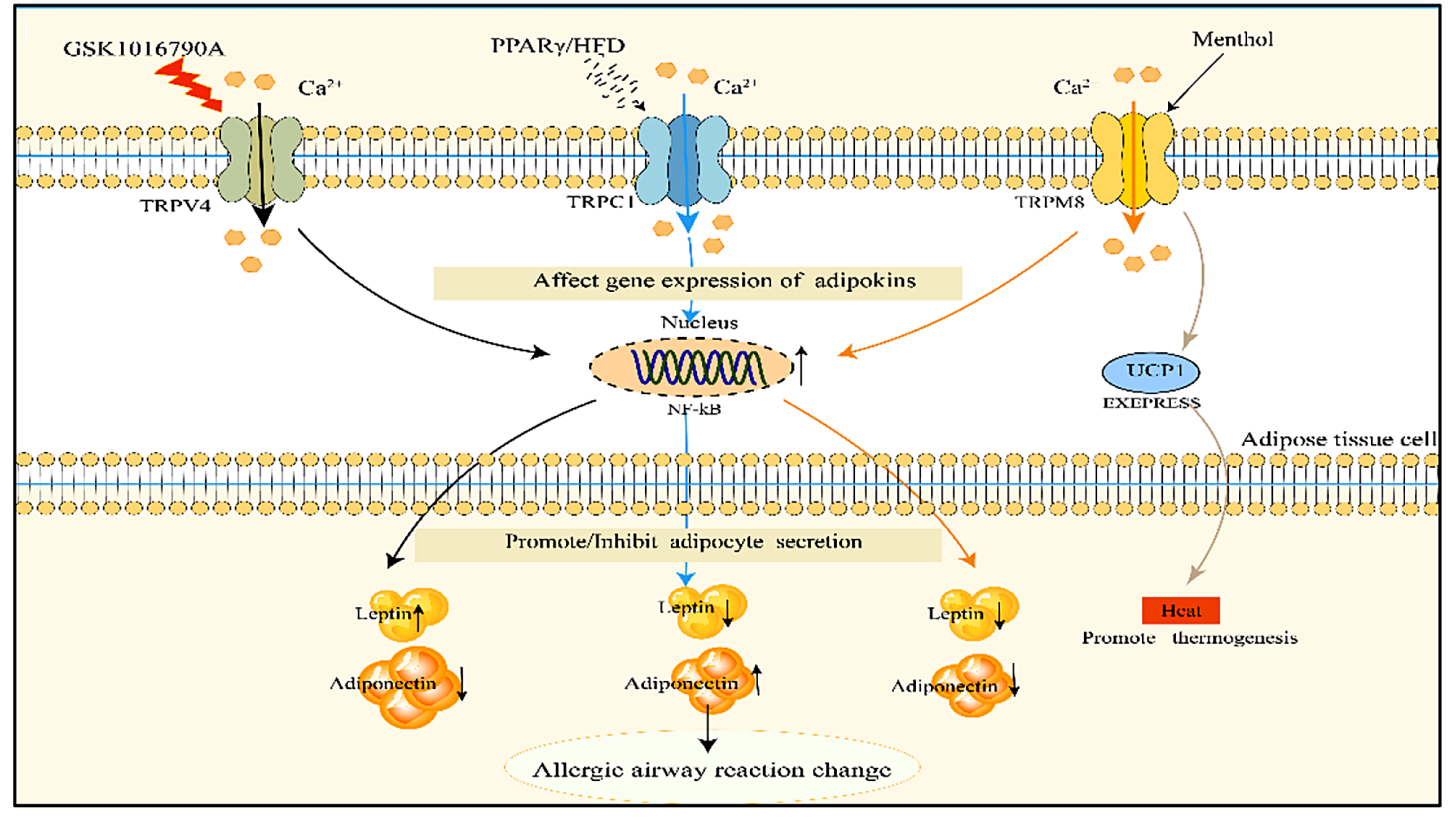
TRP channels on adipose tissue cells participate in the mechanism of obese asthma

**Fig. 3:**
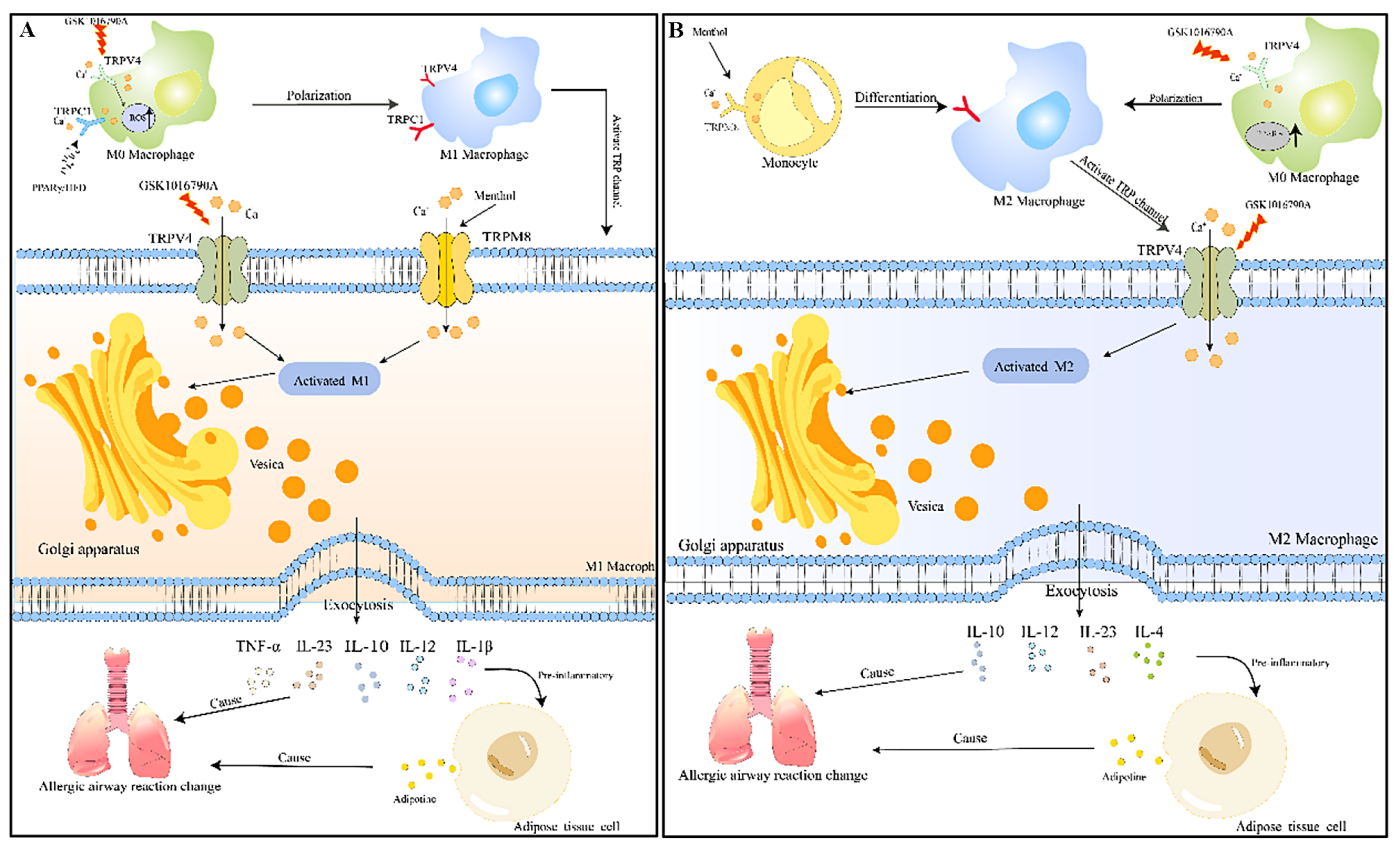
**A** TRP channels on M1 macrophages participate in the mechanism of obese asthma; **B** TRP channels on M2 macrophages participate in the mechanism of obese asthma

TRPV4 is closely associated with obesity and asthma, and to target TRPV4 for the treatment of obese asthma, the key is to find effective activators. Current studies have identified several activation pathways or substances: low osmolality and 4α-forbol-decanoate can modulate TRPV4 channels in white adipocytes, resulting in increased intracellular Ca^2+^ concentrations [94], and altering body weight in obese asthmatics by affecting energy storage in WAT will help patients improve asthma symptoms (Figs. 2, 3).

### TRPM8

In 2002, the TRPM8 receptor was successfully cloned from trigeminal sensory neurons by scientists, and it was subsequently named the menthol receptor [95]. TRPM8 is expressed not only in adipocytes but also in immune cells, thereby participating in the regulation of cytokine secretion and contributing to the inflammatory process.

The activity of TRPM8 is essential for the normal differentiation of monocytes in macrophages. Comparative gene expression analysis in samples of CD14 + monocytes returned for their macrophage differences, 3109 genes (26.6% of the expressed genes), were significantly downregulated, while 3403 genes (29.1% of the express genes) were significantly increased. It has been observed that the antagonism of TRPM8 has led to an impairment in the capacity for differentiation in macrophages [96].

Furthermore, the expression of TRPM8 in macrophages in mice has been found to determine pro- or anti-inflammatory effects mediated by TNF-α and IL-10 [97]. In mouse macrophages, the histomorphous expression of TRPM8 activates an anti-inflammatory cytokine profile and enhances phagocytosis. Conversely, the deletion of the TRPM8 gene or its pharmacological blockade induces the opposite effect.

The role of TRPM8 in asthma has been studied and confirmed. Researchers have established a mouse model of allergic asthma by exposing mice to a combination of formaldehyde at a concentration of 0.8 mg/m3 and a low temperature of 16 °C. It has been observed that simultaneous exposure to formaldehyde and low temperatures exacerbates allergic asthma. However, when the ion channels of TRPM8 and TRPA1 are blocked, inflammation levels are significantly reduced. These findings indicate that co-exposure to formaldehyde and hypothermia can worsen allergic asthma, and both TRPM8 and TRPA1 are implicated in this process [98]. In some studies, the expression of TRPM8 mRNA in BEAS 2B was examined using real-time quantitative PCR, immunofluorescence staining, and western blotting. It was discovered that the level of TRPM8 protein in asthma patients treated with bronchodilators was higher than in those without treatment [99].

Furthermore, a study conducted on mice fed a high-fat diet and treated with menthol demonstrated that the coadministration of oral menthol (at doses of 50 and 100 mg/kg) significantly prevented weight gain induced by the high-fat diet. Notably, menthol is known to be the most potent activator of TRPM8. During this process, changes in leptin and ADPN levels, which are associated with airway inflammation, were observed. Leptin levels decreased, while ADPN levels increased with increasing menthol dosage [100]. Activating TRPM8 on adipose tissue cells can control the secretion of leptin and ADPN, reduce the levels of adipocytokines that promote airway inflammation, and help suppress asthma. Activating TRPM8 on macrophages can secrete TNF- α and cytokines such as IL-10, which in turn promote macrophage phagocytosis and help alleviate airway hyperresponsiveness. TRPM8 is primarily activated by temperature, menthol, analogues, voltage, and changes in extracellular osmolarity [101]. It can be sensed as a harmless low-temperature stimulus within the range of 18–25 °C. Hence, all of these agonists may have potential therapeutic implications for obese asthma. However, research is necessary to determine the specific role of TRPM8 in other cell types (Figs. 2, 3).

### TRPC1

Similar to TRPV4 and TRPM8, TRPC1 is highly expressed in adipose tissue cells and immune cells. As a member of the TRP family, TRPC1 functions as a non-selective cation channel that is regulated by the concentration of Ca^2+^.

The generation of the M1 phenotype is strictly dependent on the STAT1 activated by TRPC1 /NF- κ. The whole-genome analysis of the B pathway also showed that the majority of M1-related genes in cells obtained from TRPC1 were reduced, and the majority of M2-related genes were significantly increased. Interestingly, the lack of TRPC1 gene expression effectively prevented the development of the M1 functional phenotype but had no impact on the M2 phenotype [102]. TRPC1-mediated Ca^2+^ influx occurs during IFN-γ activation or bacterial infection, leading to the polarization of macrophages towards the M1 phenotype. Remarkably, the deletion of TRPC1 completely inhibits the activation of M1 cells, suggesting that targeting TRPC1 could be a promising approach for the treatment of inflammatory diseases. The activation of the TRPC1 channel on macrophages is beneficial for regulating the function of TRPC1 channel and adipocytes around adipose tissue, promoting the secretion of inflammatory factors by macrophages, which enter the lungs and cause airway hyperresponsiveness. TRPC1 may act indirectly as an agonist, leading to obese asthma. Therefore, in obese asthma patients, the activity of the TRPC1 channel will determine the development of the disease.

TRPC1 has also been implicated in the inflammation associated with asthma. The expression of TRPC1 in epithelial cells was assessed using real-time quantitative PCR and immunohistochemical methods. TRPC1 contributes to abnormal Ca^2+^ signalling in response to receptor stimulation and mechanical stimuli, which in turn leads to airway remodelling. These findings provide a theoretical basis for the association among TRPC1, obesity, and asthma [103].

Furthermore, TRPC1 is expressed in various immune cells. In mice deficient in TRPC1, there was a significant reduction in allergen-induced lung leukocyte infiltration, accompanied by a diminished T helper type 2 (Th2) cell response [104]. Studies have demonstrated that TRPC1, which serves as a crucial store-operated calcium entry channel in AT, plays an important role in revealing the mechanisms underlying obesity and obese asthma. It affects the differentiation of adipose tissue cells and contributes to fat deposition. Notably, TRPC-/- mice of all age groups exhibited significantly reduced serum leptin and ADPN levels, and they exhibited weight gain as they aged [105] (Figs. 2, 3).

### Other TRP channels

In addition to the three TRP channels mentioned above, there are other TRP channels that, although their role in the inflammatory process of asthma and obesity has not been clearly researched, hold research value and warrant further exploration.

TRPV2 is a temperature-sensitive receptor that is activated when the temperature exceeds 52℃. Currently, it is primarily expressed in adipose tissue cells and is involved in adipose tissue thermogenesis. Studies on TRPV2 knockout mice have revealed abnormal expression of various adipose tissue thermogenesis. This is because the TRPV2-mediated influx of Ca^2+^ is involved in the induction of heat-producing genes following adrenergic receptor activation [106]. Therefore, compared to WT mice, brown adipocytes isolated from TRPV2 knockout (TRPV2KO) mice showed reduced expression of the heat-producing genes Ucp1 and Pgc1α. TRPV2 knockout mice exhibited impaired adipose tissue function. Other studies have also demonstrated that TRPV2KO mice exhibit cold intolerance, increased adipocyte size, and fat accumulation, providing evidence that TRPV2 is functionally expressed in BAT [107]. The application of certain TRPV activators in reducing obesity is crucial. Recent research has shown that cannabidiol can activate mitochondrial autophagy [108], and enteropathogenic *Escherichia coli* (EPEC) can induce Ca^2+^ influx [109].

The TRPA channel is an Ir non-selective Ca^2+^ channel. In mammals, the only member of the TRPA family is TRPA1. TRPA1 is widely expressed in both nerve and non-nerve cells [110]. The most well-known agonist for TRPA1 is cinnamaldehyde, which is derived from cinnamon. Recent research has identified new agonists for TRPA1, including subtilisal, anisaldehyde, and tiglic aldehyde [111]. In relation to adipose tissue, which is closely linked to obesity-induced asthma, TRPA1 has also been found to be involved in adipose tissue thermogenesis. Cinnamaldehyde has been shown to induce wat browning and activate BAT in mice fed a high-fat diet, leading to increased glucose utilization and reduced fat accumulation [112]. Studies have also discovered that allicin garlic juice (ARGJ), a novel dietary agonist, can prevent metabolic complications caused by a high-fat diet in mice and restore the balance of intestinal hormone levels after meals [113].

In addition to the TRP channels that have been implicated in the inflammatory process of obesity-induced asthma, there are other TRP channels with research significance, such as TRPM7. Additionally, the absence of TRPM4 affects macrophage infiltration and increases the levels of monocytes [114]. These channels are likely to become important targets for the future treatment of obesity-induced asthma, and further research and exploration are warranted.

It is not difficult to find that if we want to treat obese asthma through the association between obesity and asthma inflammation, TRP channels on immune cells and adipocytes will play a crucial role. Among the numerous TRP channels, we have chosen representative ones, among which TRPV4 is particularly prominent because it is highly expressed in macrophages and participates in IFN-γ [115]. We hope that future research can focus on exploring effective activators or inhibitors of TRPV4, which are harmless to the human body and can be modified as new targeted drugs for treating obese asthma. In addition, we listed other TRP channels, such as TRPV2 [116], which have not been fully studied to prove a clear association with obesity-induced asthma. However, other studies have found that they are crucial in the onset mechanisms of obesity and asthma. We define them as potential TRP channels, and further research is needed in the future to discover whether they have potential as targets.

Numerous TRP channels play a role in adipose tissue and immune cells, specifically in controlling the secretion of leptin, adiponectin, and various inflammatory factors by activating or inhibiting these TRP channels. These factors can enter the airways and cause airway inflammation such as asthma. If suitable activators are found to control TRP channels, the secretion of pro-inflammatory cytokines, their levels in the airway, and the risk of airway inflammation can be reduced, thereby preventing the occurrence of obesity-induced asthma. Treating obese asthma through TRP channels may become effective.

## Achievements and lacks

Compared with other studies, this review not only explains the pathogenesis of obese asthma, but also emphasizes for the first time the role of TRP channels in the treatment of obese asthma, with a focus on inflammation, which has a certain breakthrough. The research focuses on several TRP channels with research potential, especially TRPM8, TRPC1, and TRPV4, while also taking into account the TRP channels mentioned in general research. However, compared with some other studies related to TRP channels, there are still some lacks, such as a lack of introduction and discussion of niche TRP channels. Secondly, compared with similar articles, there is a lack of specialized experiments to evaluate the role of TRP channels. Finally, the manuscript emphasizes the role of macrophages, and compared with other immune related studies, there is insufficient description of other immune cells.

## Conclusion

This article systematically explores the link between obesity and asthma, focusing specifically on the inflammatory mechanism. It emphasizes the potential breakthrough in the treatment of obese asthma by targeting TRP channels associated with inflammation. Given the complexity and uniqueness of obese asthma, we have identified three TRP channels – TRPV1, TRPM8, and TRPC1 – and examined their role in immune cells, particularly macrophages. Consequently, it is possible that targeting TRP channels can effectively address obese asthma. Furthermore, additional TRP channels with therapeutic potential will be identified through extensive research. This highlights the need for future studies to investigate how to regulate TRP channels to control inflammation in obese asthma, identify effective activators with minimal side effects, and explore other members of the TRP family as potential targets.

## Data Availability

All data are incorporated into the article and its online supplementary material.

## Abbreviations

^18^F-FDG: ^18^F-fluorodeoxyglucose
ADP: Adiponectin
ARGJby Argj: Allicin garlic juice
AT: Adipose tissue
ATM: Ataxia telangiectasia mutated
ATP: Adenosine triphosphate
B cell: Bursa-dependent lymphocytes
BAT: Brown adipose tissue
BSF-2: B-cell stimulator factor 2
C/EBPα: Enhancer-bindingprotein α
EMS: Ethylmethylsulfone
EPEC: Enteropathogenic Escherichia coli
GC: Glucocorticoid
HFD: High-fat diet
IL-: Interleukin
ILC2: Type 2 innate lymphocytes
INF-γ: Interferon γ
M(M1、M2)M (M1 and M2): Macrophages
MC: Mast cells
MCP-1: Monocyte chemotactic protein-1
MCT: Trypsin-positive mast cells
MCTC: Chymase-positive mast cells
NCR1: NK cell-activating receptor
NK cell: Natural killer cells
NO: Nitrogen monoxide
PET-CT: Computed tomography (PET-CT)
Pgc1α: Peroxisome proliferator-activated receptor γ Co activation factor 1-α
PPARγ: Peroxisome proliferator-activated receptor γ
ROS: Reactive oxygen species
SARP: Severe Asthma Study
SOCE: Store-operated calcium entry
ST2: Growth STimulation expressed gene 2
T cell: Thymus-dependent lymphocytes
TGF-β: Transforming growth factor-β
Th-: T helper cell
TNF-α: Tumor necrosis factor-α
Tregs: Regulatory T cells
TRP: Transient receptor potential
TRPA: Transient receptor potential ankyrin
TRPC: Transient receptor potential canonical
TRPM: Transient receptor potential melastatin
TRPMLTRPML is: Transient receptor potential mucolipin
TRPN: Transient receptor potenti NO-mechano-potential
TRPP: Transient receptor potential polycystin
TRPV: Transient receptor potential vanilloid
UCP1: Uncoupling protein 1
WAT: White adipose tissue
WT: Wild-type
